# Prenatal folic acid use associated with decreased risk of myelomeningocele: A case-control study offers further support for folic acid fortification in Bangladesh

**DOI:** 10.1371/journal.pone.0188726

**Published:** 2017-11-30

**Authors:** Vijaya Kancherla, Md Omar Sharif Ibne Hasan, Rezina Hamid, Ligi Paul, Jacob Selhub, Godfrey Oakley, Quazi Quamruzzaman, Maitreyi Mazumdar

**Affiliations:** 1 Department of Epidemiology, Rollins School of Public Health, Emory University, Atlanta, Georgia, United States of America; 2 Dhaka Community Hospital, Dhaka, Bangladesh; 3 Bangladesh Medical College, Dhaka, Bangladesh; 4 Jean Mayer USDA Human Nutrition Research Center on Aging, Tufts University, Boston, Massachusetts, United States of America; 5 Department of Neurology, Boston Children’s Hospital, Boston, Massachusetts, United States of America; 6 Department of Environmental Health, Harvard T.H. Chan School of Public Health, Boston, Massachusetts, United States of America; Seoul National University, REPUBLIC OF KOREA

## Abstract

Neural tube defects contribute to severe morbidity and mortality in children and adults; however, they are largely preventable through maternal intake of folic acid before and during early pregnancy. We examined the association between maternal prenatal folic acid supplement intake and risk of myelomeningocele (a severe and common type of neural tube defect) in the offspring. We performed secondary analysis using data from a case-control study conducted at Dhaka Community Hospital, Bangladesh between April and November of 2013. Cases and controls included children with and without myelomeningocele, respectively, and their mothers. Cases were identified from local hospitals and rural health clinics served by Dhaka Community Hospital. Controls were selected from pregnancy registries located in the same region as the cases, and matched (1:1) to cases by age and sex.

Myelomeningocele in the offspring was confirmed by a pediatrician with expertise in classifying neural tube defects. Maternal prenatal folic acid supplement intake was the main exposure of interest. We estimated crude and adjusted odds ratios (OR) and 95% confidence intervals (CI) using conditional logistic regression analysis. There were 53 pairs of matched cases and controls in our study. Overall, 51% of case mothers reported using folic acid supplements during pregnancy compared to 72% of control mothers (*p* = 0.03). Median plasma folate concentrations at the time of study visit were 2.79 ng/mL and 2.86 ng/mL among case and control mothers, respectively (*p* = 0.85). Maternal prenatal folic acid use significantly decreased the odds of myelomeningocele in the offspring (unadjusted OR = 0.42, 95% CI = 0.18–0.96). The association was slightly attenuated after adjusting for maternal age at the time of pregnancy (adjusted OR = 0.43, 95% CI = 0.18–1.02). Our study confirms the protective association between maternal prenatal folic acid supplement use and myelomeningocele among children born in Bangladesh. Our findings point to an overall low folic acid supplement use and low plasma folate concentrations among women of reproductive age in Bangladesh. Mandatory fortification of staple foods with folic acid can address low folate status among women of child-bearing age, and prevent child morbidity and mortality associated with myelomeningocele in Bangladesh.

## Introduction

Neural tube defects are serious birth defects characterized by incomplete development of the brain, spinal column, and spinal cord. They occur at about 3 to 4 weeks of gestation, when most women are unaware of their pregnancies. These birth defects contribute to neonatal, infant, and child mortality [1,2], and result in severe and life-long disabilities among those who survive [3]. Neural tube defects are associated with increased medical care costs, as well as loss of human potential, due to disability and death [4–6].

Bangladesh is believed to have a high rate of neural tube defects, although current national population-based prevalence estimates are lacking in the country. The March of Dimes Global Report on Birth Defects, using available data from surveillance and birth defects registries, modelled the average worldwide birth prevalence of neural tube defects to be 2.4 per 1000 live births [7]. In comparison, the report’s extrapolated data for Bangladesh indicated that the birth prevalence of neural tube defects in the country was 4.7 per 1000 live births, almost twice as high as the average global prevalence [7].

Maternal intake of folic acid before and during early pregnancy is a proven intervention for preventing a majority of cases of neural tube defects [8–10]. Despite recommendations from the World Health Organization (WHO) for the use of folic acid supplements among women of childbearing age [11], several studies have revealed low prevalence (~20%) of prenatal folic acid supplement intake in Bangladesh [12,13]. Bangladesh also has a high prevalence of malnutrition and micronutrient deficiencies, including folate deficiency, hindering optimal birth outcomes among babies born to malnourished mothers [14,15]. According to the Food Fortification Initiative (http://www.ffinetwork.org), Bangladesh does not currently have a national program for mandatory folic acid fortification for staple grains (wheat, maize, rice).

Although the role of folic acid in the prevention of neural tube defects is well- established in developed regions of the world, studies examining the magnitude of this association are lacking in Bangladesh. Given the country’s high prevalence of folate deficiency [16] and burden of neural tube defects [7], it is especially important to understand this association, and apply insights gained to ensure healthy lives and promote well-being, as proposed in the 2030 health-related Sustainable Development Goals for Bangladesh [17]. We hypothesized that maternal intake of folic acid supplements during pregnancy decrease the risk of myelomeningocele, a common and severe type of neural tube defect, in the offspring.

## Materials and methods

We used data from a case-control study conducted in the communities served by Dhaka Community Hospital in Bangladesh between April and November 2013, to assess the association between maternal prenatal folic acid supplement intake and myelomeningocele in the offspring. The original study was designed to examine the associations between environmental arsenic exposure and myelomeningocele risk, and no main effect of arsenic on myelomeningocele was observed [18].

The design, inclusion criteria, and recruitment strategy for the original study have been described previously [18,19]. Participants for our current analysis included children with myelomeningocele (cases) and their mothers. Cases were identified through regular communication with local hospitals and rural health clinics served by Dhaka Community Hospital, a large, trust-owned health system that operates primary health care centers across Bangladesh. All cases were confirmed by a pediatrician with expertise in classifying neural tube defects, using physical examination or photographs (when a case was deceased) and including an evaluation for level of neural tube defect. Control children and their mothers were randomly enrolled from pregnancy registries in the same geographic regions. Cases and controls were matched 1:1 by the child’s sex and age within 2 months. Participation rate was 98% and 83% among case and control mothers, respectively. The reasons for refusing to participate in the study were similar between case and control mothers, including lack of interest to participate, and unwillingness to provide blood samples due to fear of sickness. The sample size was estimated *a priori* based on the original study hypothesis [18]. Trained interviewers collected information on history of maternal folic acid use to ascertain folic acid-containing supplement intake during the pregnancy. Interviewers also collected information from mothers about their medical histories, medication use during pregnancy, family history, and reproductive history. Study activities consisted of a single clinical visit during which we administered questionnaires, performed physical examinations, and collected samples. The age of the infant at the study visit, therefore, represents the age of the infant since delivery as well as the time since postpartum state of the mother.

Maternal blood samples were collected via venipuncture at the time of the interview. Blood samples were transported to Dhaka within one day of collection in an insulated container, and stored at −20°C. Plasma from the centrifuged blood samples was collected into microcentrifuge tubes and stored at −20°C. Blood/plasma samples were shipped to Harvard School of Public Health on dry ice. Folate analyses were performed at the Vitamin Metabolism Laboratory at the Jean Mayer United States Department of Agriculture (USDA) Human Nutrition Research Center at Tufts University. Total folate concentrations in the plasma samples were measured by microbial assay with the use of *Lactobacillus casei* [19]. Each plasma sample (5 μL) was serially diluted and plated in triplicate onto a 96-microtiter well plate with 150 μL of *Lactobacillus casei* growth medium. Plates were intubated overnight in a 37°C humid incubator and then absorbance was measured, which indicated microbial growth, with the use of a 96-well plate reader (PowerWave HT; BioTek Instruments Inc, Winooski, VT USA) at 595 nm. As arsenic is a major concern in Bangladesh, tests were done to check if any arsenic in plasma affected the microbial assay, including spiking 3 random samples with 5 ng/mL folic acid, and there were no inhibitory components detected in the plasma. The coefficients of variation for the assay using one plasma sample with high folate concentration and one sample with low folate concentration were 6.78% and 4.73%, respectively. For our study analysis, we used the World Health Organization’s guideline on establishing plasma folate deficiency at a cut-off value of 4.0 ng/mL (http://apps.who.int/iris/bitstream/10665/75584/1/WHO_NMH_NHD_EPG_12.1_eng.pdf Accessed on November 1, 2017).

The Human Research Committees at the Boston Children’s Hospital and Dhaka Community Hospital approved this study. Consent documents were provided to participants in Bengali and reviewed with potential participants by local study staff. All participants provided written informed consent.

### Statistical analysis

We conducted a descriptive analysis of selected child and maternal characteristics by case and control status. We tested distribution using Shapiro-Wilk Test for Normality. We used independent sample t-test to compare mean ages of case and control infants and mothers at the time of the study visit, and Wilcoxon two-sample test to compare median folate concentrations between case and control mothers. We examined the difference between maternal prenatal folic acid intake among matched case-control pairs using McNemar’s Test. We used conditional logistic regression to estimate unadjusted and adjusted odds ratios (uOR and aOR) and associated 95% confidence intervals (CI) for the association between maternal prenatal folic acid supplement use and risk of myelomeningocele in the offspring. Potential confounding due to maternal age at delivery, family history of birth defects, family history of neural tube defects, valproic acid use during pregnancy, and pre-pregnancy diabetes was considered. Co-factors were selected based on *a priori* criteria assessed from previous studies [4]. All analyses were conducted using SAS (version 9.4) for windows (SAS Institute, Inc. Cary, NC, USA).

## Results

There were 53 pairs of matched cases and controls in our study. The mean ages of case and control infants, and case and control mothers, at the time of the study visit, are presented in Table 1. Among the case infants, 60% were males, 27% were born preterm, 32% had a low birth weight (<2500 grams), 50% were delivered at home, and none had a family history of neural tube defects or other birth defects (Table 1). Almost 85% and 90% of case and control mother, respectively, reported to have received an ultrasound examination before their index pregnancy (data not shown). Except for low birth weight, no other characteristic differed significantly between the case and control infants. We compared selected characteristics between case and control mothers, and found that case mothers were four times more likely to be older (>30 years) than control mothers at the time of delivery. There were no significant differences between case and control mothers with respect to prenatal exposures to smoking and valproic acid. None of the case or control mothers reported being exposed to alcohol or pesticides during pregnancy, or having a history of pre-pregnancy diabetes (Table 1).

**Table 1:** Infant and maternal characteristics of cases (myelomeningocele) and controls.

	**Cases** **(n = 53)**		**Controls** **(n = 53)**			
	**Mean (SD)**		**Mean (SD)**		***p* value**	
Child’s age at study visit (Months)	5.7 (5.3)		7.8 (4.8)		0.03	
Mother’s age at study visit (Years)	22.2 (4.1)		24.7 (5.3)		0.01	
	**n**	**(%)**	**n**	**(%)**	**Unadjusted Odds Ratio** **(95% CI)**	**Adjusted Odds Ratio** **(95% CI)**
**Child**						
Gestational age at delivery						
Term	37	(70)	31	(59)	Reference	—
Preterm	14	(26)	17	(32)	0.64 (0.26–1.59)	
Post-term	2	(4)	5	(9)	0.33 (0.06–1.80)	
Birth weight, grams						
<2500	17	(32)	8	(15)	2.50 (0.97–6.44)	—
2500	36	(68)	45	(85)	Reference	
Place of birth						
Home	26	(49)	21	(40)	2.11 (0.82–5.44)	—
Clinic	15	(28)	9	(17)	2.36 (0.88–6.32)	
Hospital	12	(23)	23	(43)	Reference	
Delivery type						
Vaginal	30	(57)	32	(60)	Reference	—
Cesarean Section	23	(43)	21	(40)	0.86 (0.40–1.85)	
Birth Order						
First born	22	(42)	25	(47)	Reference	—
Second or higher	31	(58)	28	(53)	1.38 (0.55–3.42)	
Family history of birth defects						
No	53	(100)	53	(100)	NA	—
Yes	0	-	0	-		
Family history of NTD						
No	53	(100)	53	(100)	NA	—
Yes	0	-	0	-		
**Mother**						
Age at delivery (years)						
<20	10	(19)	14	(26)	0.95 (0.35–2.57)	1.00 (0.35, 2.84)
20–30	35	(66)	37	(70)	Reference	Reference
>30	8	(15)	2	(4)	3.88 (0.74–20.36)	2.79 (0.67, 21.29)
Prenatal folic acid supplement use						
No	26	(49)	15	(28)	Reference	Reference
Yes	27	(51)	38	(72)	0.42 (0.18, 0.96)	0.43 (0.18, 1.02)
Folic acid deficiency at study visit						
No (≥4.0 ng/mL)	18	(35)	16	(30)	Reference	—
Yes (<4.0 ng/mL)	33	(65)	37	(70)	0.80 (0.35–1.80)	
Pre-pregnancy diabetes						
No	53	(100)	53	(100)	NA	—
Yes	0	-	0	-	Reference	
Smoking during pregnancy						
No	52	(98)	53	100	NA	—
Yes	1	(2)	0	-		
Alcohol during pregnancy						
No	53	(100)	53	(100)	NA	—
Yes	0	-	0	-		
Pesticide exposure during pregnancy						
No	53	(100)	53	(100)	NA	—
Yes	0	-	0	-		
Valproic acid use during pregnancy						
No	53	(100)	53	(100)	NA	—
Yes	0	-	0	-		

CI, Confidence Interval; NA, Not Applicable; NTD, Neural Tube Defects

Frequencies and percentage may not equal total due to missing data.

**Fig 1:**
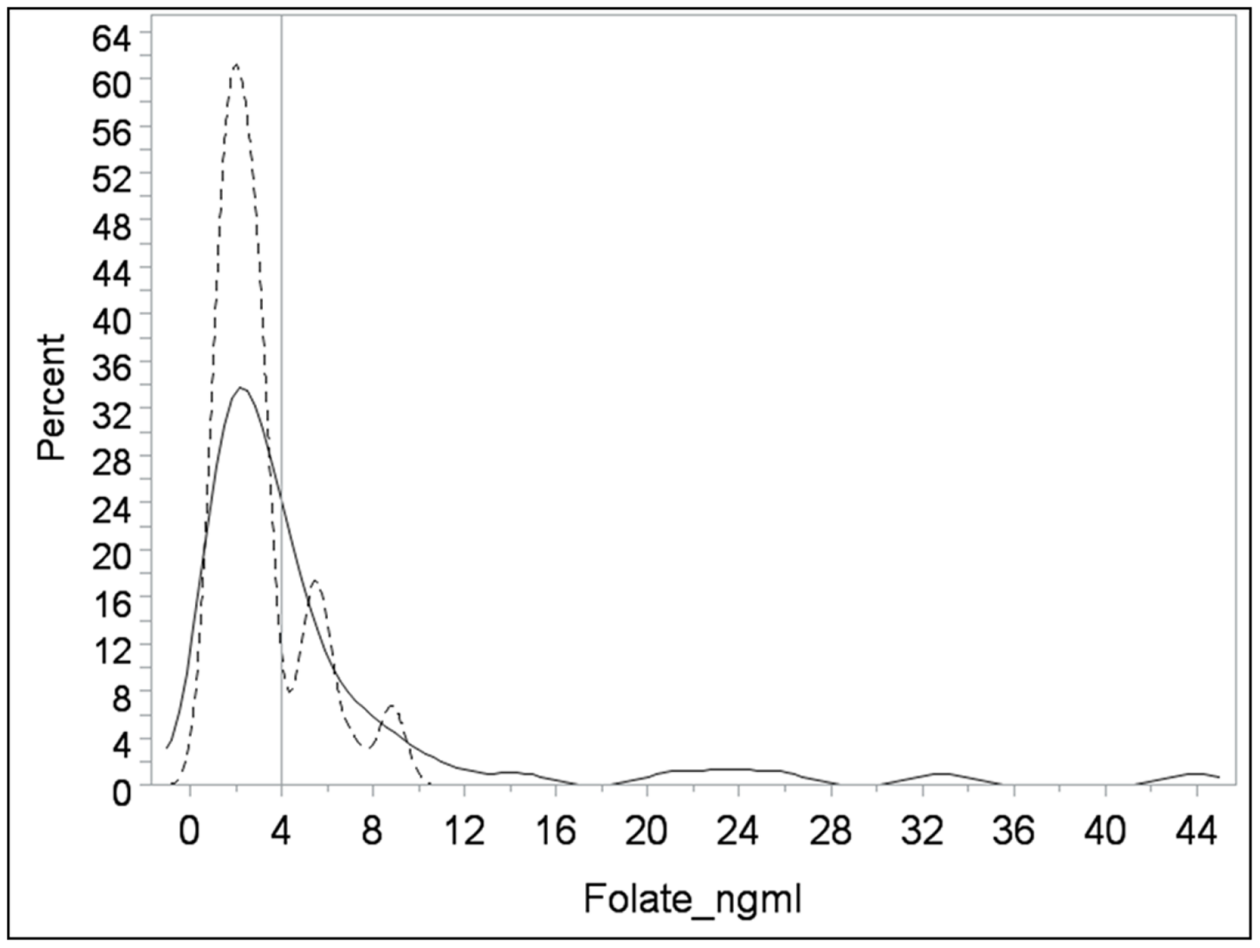
Plasma folate concentrations at the time of the study by maternal prenatal folic acid supplement intake during pregnancy. Solid black line represents the distribution of plasma folate concentrations at the time of the study visit among mothers who reported prenatal folic acid supplement intake during pregnancy. Dotted line represents the distribution of plasma folate concentrations among mothers who reported that they did not use prenatal folic acid supplements during pregnancy. The threshold for optimal folic acid concentration is represented by the straight line at 4 ng/ml.

We noted that 65% and 70% of case and control mothers, respectively, had low plasma folate concentrations at the time of the study visit (defined as a level lower than 4.0 ng/mL) at the time of the study visit (Table 1). The median plasma folate levels were 2.79 ng/mL among case mothers and 2.86 ng/mL among control mothers; however, the difference between the groups was not statistically significant (*p* = 0.85) (data not shown). Fig 1 shows the distribution of plasma folate concentrations among mothers at the time of study visit, by self-reported prenatal folic acid supplement use during pregnancy. It is clear from this figure that mothers who reported taking prenatal supplements were more likely to have higher plasma folate concentrations at the time of the study visit compared to mothers who did not report taking supplements.

Analysis of matched pairs showed that there were 27 discordant pairs, i.e., pairs in which case mothers did not take folic acid but the control took folic acid (n = 8); and pairs in which the control mother did not take folic acid but the case mother did (n = 19). This difference in prenatal folic acid intake was statistically significant among the matched case-control pairs (McNemar’s *p* = 0.03).

Results from the conditional logistic regression showed that maternal prenatal folic acid supplement use had a significant protective association with the risk of myelomeningocele (uOR = 0.42, 95% CI = 0.18–0.96) (Table 1). This association slightly attenuated after adjusting for maternal age at delivery (aOR = 0.43, 95% CI = 0.18–1.02); however, the magnitude of protective effect persisted. We were unable to examine potential confounding due to family history of neural tube defects, maternal pre-pregnancy diabetes, and prenatal exposure to valproic acid use, which are known risk factors for myelomeningocele, due to null exposures among case or control mothers.

## Discussion

Our study is the first case-control study to report the association between maternal prenatal folic acid supplement use and myelomeningocele in Bangladesh. We found a strong protective association, with up to 60% reduction in the risk of myelomeningocele from maternal prenatal folic acid use. There was a significant difference among case and control mothers with regard to the prevalence of folic acid supplement use during pregnancy. Up to three-quarters of both case and control mothers had low plasma folate concentrations at the time of the study visit. The protective findings of prenatal folic acid supplementation in our study, and the low use of folic acid supplementation by women of reproductive age, strongly suggest that folic acid fortification of the food supply would be a more effective preventive strategy for major and common neural tube defects in Bangladesh.

Our results are consistent with findings from other case-control studies conducted in countries that did not have fortification programs at the time the study was conducted. A case-control study in Japan, a country that currently has no required folic acid fortification of staple foods showed maternal periconceptional folic acid-containing supplement intake reduced the risk of spina bifida in offspring by approximately 50% during two different study periods (study years 2001–2006: aOR = 0.48, 95% CI = 0.23–0.96; and study years 2007–2012: aOR = 0.53, 95% CI = 0.34–0.84) [20].

Before the United States implemented mandatory folic acid fortification of staples including wheat and maize (prior to 1998), a protective association was noted between maternal periconceptional multivitamin intake and the risk of spina bifida among births in Metropolitan Atlanta with an estimated relative risk of 0.37 (95% CI = 0.19–0.70) [21]. Post- folic acid fortification studies in the United States failed to demonstrate a protective effect of extra folic acid in multivitamin supplement pills on the risk of neural tube defects, where mandatory folic acid fortification has been attributed to near complete prevention of spina bifida [22].

The World Health Organization has recently released guidelines on optimal blood folate concentrations among women of reproductive age to prevent neural tube defects [23]. According to these guidelines, one can achieve maximum prevention of neural tube defects at a population level when the red blood cell concentrations of women of reproductive age are above 400 ng/mL (or 906 nmol/L). Concentrations below this threshold indicate folate insufficiency (i.e. concentrations that increase the risk of having an NTD-affected pregnancy). No established threshold exists for plasma folate concentration. However, folate deficiency has been established at a concentration of less than 4.0 ng/mL (<10 nmol/L) [24]. Our study, as well as other studies conducted recently in Bangladesh, indicate marked folate deficiency among women in Bangladesh [25,26]. We used plasma folate in our studies as this is measure widely used in Bangladesh.

Our findings have limitations as they were based on a retrospective, observational study design. There is a potential for recall bias in responses related to pregnancy exposures and medical history. Plasma samples for folate assessments were drawn after the index pregnancy and not in the first trimester. Information on folic acid supplement use before conception was not collected. Further, the exact time of use and dosage of folic acid supplements taken during pregnancy was not inquired. However, we noticed that the mean plasma folate concentrations were higher at the time of study visit among women who reported folic acid use compared to their counterparts. The strong correlation between maternal report of prenatal folic acid supplement use and plasma folate concentrations validates maternal responses in the study, though it is important to reiterate that the plasma was collected many months after the pregnancy was completed and therefore represents current plasma folate and not folate levels during early pregnancy.

There were many strengths to our study. We had a high diagnostic accuracy and specificity in all myelomeningocele phenotypes, which were confirmed by an expert pediatrician. Controls were selected randomly from the same region as the cases. Maternal interviews were conducted by trained interviewers, and several important risk factors were assessed that allowed for a thorough analysis of potential confounders.

Bangladesh does not require folic acid fortification of staple foods. Instead, the country has invested in the national prenatal care program that promotes supplemental folic acid intake among women of reproductive age, which is largely determined by individual health behaviors and adherence patterns [27]. Our case-control study highlights the protective effect of folic acid supplement use alone on myelomeningocele, and possibly other neural tube defects. Bangladeshi women have also been found to be consuming a diet that does not provide an adequate daily dose of folate [17].

One would expect that case-control studies in areas with mandatory folic acid fortification would not suggest an additional protective effect from folic acid in supplements [28,29]. This can be attributed to improved blood folate concentrations gained through fortification, therefore yielding no additional protective effect from folic acid supplements. Bangladesh does not yet have a folic acid fortification mandate; thus, as expected, we were able to demonstrate a protective effect from folic acid supplements on the risk of myelomeningocele.

Data from several countries demonstrate that the mandatory fortification of staple foods is a more effective public health strategy for neural tube defects prevention than folic acid supplementation alone [30]. Folic acid supplementation program alone is less effective as a mass public health intervention because of several reasons, including unplanned pregnancies, lack of preconception care (aiming at the critical period for development of neural tube defects at 28 days after conception), delay in receiving prenatal care early in pregnancy, lack of knowledge or access to folic acid, and achieving compliance in supplement intake behaviors of women. In the backdrop of aforementioned conditions, the World Health Organization recommends folic acid fortification for countries where there is a high prevalence of malnutrition and unplanned pregnancies, such as Bangladesh [24]. Findings from our study suggest that if Bangladesh were to require folic acid fortification of a widely eaten and centrally processed food, such as rice or wheat, women would benefit from availability of folic acid from fortified foods at the right time, i.e., prior to the conception and during early pregnancy, the period when the neural tube develops in the embryo. At the same time, supplementation programs can be rolled out in areas where mandatory fortification cannot reach populations of Bangladesh. In combination, the two interventions would provide a complimentary and fast-paced solution in addressing neural tube defects reduction in Bangladesh. Preventing the mortality and morbidity associated with neural tube defects through folic acid fortification has the potential to help Bangladesh to move towards achieving the 2030 Sustainable Development Goals.
